# Creating Inclusive Research Through a Culturally Aware Data Collection Site

**DOI:** 10.1002/alz.087577

**Published:** 2025-01-09

**Authors:** Hector Salazar, Jennifer H Lingler, Carola A. Ferrer Simó, Fabu P. Carter, James Bester, Lytonia Floyd, Tracy SmithBA, Annik Dupaty, Carey E. Gleason

**Affiliations:** ^1^ University of Wisconsin‐Madison, Madison, WI USA; ^2^ University of Pittsburgh Alzheimer’s Disease Research Center (ADRC), Pittsburgh, PA USA; ^3^ Wisconsin Alzheimer’s Disease Research Center, University of Wisconsin School of Medicine and Public Health, Madison, WI USA; ^4^ Wisconsin Alzheimer’s Disease Research Center, University of Wisconsin‐Madison School of Medicine & Public Health, Madison, WI USA; ^5^ Wisconsin Alzheimer’s Disease Research Center, Madison, WI USA; ^6^ Wisconsin Alzheimer's Disease Research Center, University of Wisconsin School of Medicine and Public Health, Madison, WI USA

## Abstract

**Background:**

Ethnic and racial diversity in clinical research is critical for developing generalizable treatments and caregiving strategies. Barriers to participation among persons from underrepresented groups (URG) are systemic in clinical research. To increase URG research participation, we designed a community‐based data collection site where study participants complete full research visits.

**Method:**

For an Alzheimer’s disease study, we established the first community‐based data collection site in Madison, WI, forging connections with Black community leaders and campus outreach staff. The site is proximal to diverse neighborhoods, public transportation, and is a hub for multi‐ethnic businesses. We rent space in the South Madison Partnership Office, an established community outreach center. This has allowed us to cultivate a consistent presence in the Black community, which evolved into a full data‐collection site. Staff include research specialists, community liaisons, nurse practitioners, and clinicians who are culturally‐aware and members of the Black community. The site is decorated with art from local Black and Indigenous artists, and food served is from a local Black‐owned business.

**Result:**

We established proof of concept by successfully launching a fully‐equipped research site in the community, used for memory testing, biometric readings, blood draws, and one‐on‐one conversations with clinicians. Initial comments suggest participants prefer this comfortable, familiar setting over that of a hospital setting.

**Conclusion:**

Researchers can use culturally‐aware and convenient data collection sites. Our site prioritizes the needs of participants toward the goal of achieving representation of racialized groups in research. This model has potential for broad implementation across aging and biomedical research.

